# Measures of Implicit Gender Attitudes May Exaggerate Differences in Underlying Associations among Chinese Urban and Rural Women

**DOI:** 10.5334/pb.308

**Published:** 2016-01-29

**Authors:** Zheng Jin, Andrew M. Rivers, Jeffrey W. Sherman, Ruijun Chen

**Affiliations:** 1 Institute of Educational Science, Zhengzhou Normal University, China; 2 Department of Psychology, University of California, Davis, USA

**Keywords:** implicit attitude, gender attitude, self-regulation, automatic associations, multinomial model

## Abstract

The oppression of women in rural China is more severe than in urban China, not only because the two areas differ in terms of social hierarchy, but also because urban women are more likely to fight against their subordination, which is endorsed by conventional social views on gender. To independently assess these relationships, we applied the Quadruple Process model to measure the processes underlying implicit gender attitudes in a sample of urban and rural females. The results indicated that the urban women had higher in-group favoritism than did the rural women. Application of the Quad model, however, showed that pro-women associations were similarly activated among urban and rural women, but that women in rural settings more effectively inhibited activated associations. Differences in inhibition, rather than in activated associations, appear to account for the less favorable attitudes among rural women. Thus, the differences in attitudinal responses among urban and rural women exaggerate the differences in underlying evaluative associations with respect to gender and conceal differences in self-regulating the expression of those associations.

During the past several thousand years, few countries in the world have been influenced by patriarchal ideology as much as China, and women in China experience the most severe discrimination (for a review, see Banister, 2004). The oppression of women is reflected in many aspects of real life in China and is worse in rural China (Attane & Croll, 2001; Croll, 1985; Jacka, 1997, 2006; To & Tam, 2014). In contrast to men, in-group favoritism (i.e., a pattern of favoring members of one’s own-group over out-group members) among women is undoubtedly conflicted by their subordination (Sidanius & Pratto, 1999). Relevant studies on feminism have suggested that pro-women ideology (e.g., valuing women) has a hierarchy-attenuating role in mediating this conflict and in achieving gender equality (Bartky, 1975; Downing & Roush, 1985; Henderson-King & Stewart, 1994; Offen, 1988). In other words, women are expected to be more likely to fight against oppression if they value themselves more or, at the very least, possess greater in-group favoritism. The development of social civilization and changes in government policy have caused conventional attitudes that support traditional ideology to be replaced by egalitarian ideas that support gender equality. This progress has occurred to a significantly greater extent in urban China than in rural China (Whyte, 2010). As a result, urban women may have more in-group favoritism than do rural women, which in turn might explain why women in rural China are hesitant to confront oppression. Indeed, consistent with this account, a number of Chinese indigenous studies showed that urban people tend to be less misogynistic than rural people (e.g., Fan, 2003).

However, questions remain as to whether the gender equality associated with urbanization is related to more in-group favoritism among urban women. In considering this question, it is important to note that the term “attitude” (e.g., in-group favoritism, in this study) is traditionally defined in most social psychology research as the behavioral outcomes of either explicit (e.g., self-reported responses) or implicit (e.g., Implicit Association Tasks, IAT, Greenwald, McGhee, & Schwartz, 1998) measures. It is widely believed that implicit measures of attitudes are more indicative of the underlying and automatically activated mental associations underlying attitudes than are explicit measures of attitudes (Fazio & Towles-Schwen, 1999; Greenwald et al., 1998). However, considerable evidence shows that responses on neither explicit nor implicit measures are isomorphic with the underlying evaluative associations that instigate those responses (e.g., Sherman et al., 2008). That is, evaluative measures might not reflect underlying associations due to the operation of parallel mental processes that translate the associations into behavioral responses (Fazio, 1990; Gawronski & Bodenhausen, 2007; Sherman et al., 2008). Therefore, we refer to behavioral biases as “attitudes” in the common vernacular while differentiating this term from evaluative associations.

One important reason for independently assessing the operation of activated associations and self-regulation is that a growing body of research has shown that self-regulatory processes interact with activated associations to determine responses on both implicit and explicit measures of attitude (for review, see Calanchini & Sherman, 2013). In the case of anti-aging bias, for example, similarities in IAT latencies concealed differences in both negative-old associations and the ability to self-regulate one’s behavior (Gonsalkorale, Sherman, & Klauer, 2014). In particular, both younger and older adults displayed similar levels of antiaging bias on an Implicit Association Test (IAT) as measured with response latencies. However, multinomial modeling technique that involves estimating hypothetical parameters that represent the probabilities of unobservable cognitive events revealed that negative-old associations were less influential among older than younger adults and at the same time, older adults were less able to self-regulate their behavior while performing the task. Thus, less biased associations among the elderly were countered by a weaker ability to control the influence of those associations. A number of other findings demonstrate the joint and sometimes opposing influences of activated associations and the ability to control them (Calanchini & Sherman, 2013; Sherman et al., 2008).

These results make clear that, to understand the factors that influence gender attitudes (e.g., in-group favoritism), both the biased associations that come to mind and the self-regulatory processes that translate those associations into attitudinal responses must be examined. Specifically, differences in the expressed attitudes of urban and rural women may reflect differences in underlying evaluative associations, differences in regulating the influence of those associations, or both. One tool that has been developed, specifically, to measure both evaluative associations and the extent to which they are regulated is the Quadruple Process model (Quad model; Conrey et al., 2005; Sherman et al., 2008).

The Quad model proposes that responses on implicit measures such as the IAT reflect the operation of four qualitatively distinct processes: activation of associations (AC), detection of correct responses (D), overcoming bias (OB), and guessing (G). The AC parameter refers to the degree to which biased associations are activated when perceiving a stimulus. The D parameter reflects participants’ ability to detect correct task responses. Sometimes, activated associations conflict with detected correct responses. For example, on trials in which female names are associated with negative words, activated associations between female names and positivity conflict with detection. In this case an OB process mediates the conflict. As such, the OB parameter refers to inhibitory processes that prevent activated associations from influencing behavior when they conflict with detected correct responses. Finally, the G parameter estimates general response tendencies that occur when no activated associations direct behavior and correct responses are not detected.

Past work suggests that those living in urban areas of China are more favorable to conceptions of gender equality (e.g., Whyte, 2010). Based on this work, we predicted that women living in urban areas would express more positive implicit evaluations towards their in-group than would women living in rural areas. However, we hypothesized that differences between urban and rural women might arise for two reasons: 1) it might be the case that the underlying in-group associations differ or 2) it might be the case that associations do not differ but instead that urban and rural women differ in the extent to which they control the expression of positive in-group associations. In the present studies, we first sought to assess whether urban and rural women differ in the positivity of their implicit in-group evaluations. Then, we applied the Quad model to independently estimate the extent to which urban and rural women might differ in their underlying in-group associations (AC) and/or differ in the extent to which they control the expression of their in-group associations (OB). As there were competing possibilities for the cognitive processes that might underlie evaluative group differences, a second replication study was conducted to increase confidence in findings from the initial investigation.

## Study 1

### Method

**Participants.** Participants were 248 (123 women, 125 men, *M_age_* = 22.7 years, age range: 18–26 years) undergraduate students who visited the Psychophysics Lab of Zhengzhou Normal University during the Autumn Semester of 2014. A total of 117 female students who provided us with sociodemographic information on registered residence were selected for analysis (64 urban females, 53 rural females).

Urban areas refer to cities above prefectural-level. Suburban counties are defined as rural areas (Chan, 1994). In China, the suburban counties, being predominantly agricultural in nature, are not urban, except for the designated towns in these counties. For the composite index of urban and rural areas, this study refers to the Comprehensive Evaluation and Grading Report on China Urban Scientific Development (2008-2013).

**Materials and Procedure.** Informed consent was obtained from each participant before the testing. After providing demographic information, participants completed the gender version of the Implicit Association Task (Greenwald, Nosek, & Banaji, 2003). In the IAT, participants used two keys to categorize 16 target words (eight male names and eight female names) and 14 evaluative words (seven positive and seven negative) (see **Appendix B**). They were instructed to make their classifications as quickly and accurately as possible. They first completed two 20-trial practice blocks, in which they discriminated positive from negative words, and male from female names. The third and fourth blocks were critical blocks consisting of 20 and 40 trials, respectively. Participants were instructed to press one key whenever they saw a name of a male or a negative word and another key whenever they saw a Female name or a positive word. The keys used to categorize male and female names were switched in the remaining blocks. The fifth block was a practice block in which participants discriminated male from female names. In the last two blocks, female names shared a response key with the negative words and male names shared a response key with positive words. Participants who respond more quickly when female names share a key with positive words (compatible trials) than when they share a key with negative words (incompatible trials) are thought to have a preference for females relative to males. Category labels remained on the top left and right of the screen throughout the task, while stimuli appeared in the center of the screen. A red “X” appeared whenever participants made an error, and they were required to correct it before moving on to the next trial. The order of the critical blocks was counterbalanced across participants.

### Results and Discussion

IAT effects were computed using the D-algorithm described by Greenwald, Nosek, and Banaji (2003). In this study, the D score was calculated by subtracting male/good + female/bad RTs from male/bad + female/good RTs (reaction time) so that negative D scores reflect favoritism for females’ own gender. The overall D effect is –.45, indicating preference for female over male gender. Although the sample in this study was selected according to their reported registered residence, which may not reflect the place where they had lived longest, analysis showed that in-group favoritism was greater for the urban women (*M* = –.58, *SE* = .04) than for the rural women (*M* = –.29, *SE* = .05), *t* (115) = 4.99, *p* < .001.

**Figure 1:**
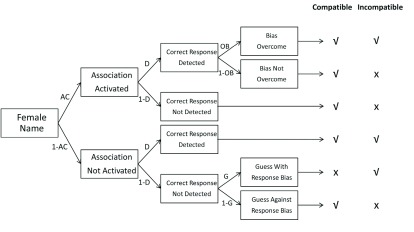
The Quadruple process model (Quad model). Each path represents a likelihood. Parameters with lines leading to them are conditional upon all preceding parameters. The table on the right side of the figure depicts correct (√) and incorrect (X) responses as a function of process pattern and trial type (for ‘‘female’’ targets). The guessing bias in this figure refers to guessing with the positive (pleasant) key (Adapted from Gonsalkorale et al., 2014).

The correct responses and errors from the IAT were then modeled with the Quad model (see Gonsalkore et al., 2009; 2014). The structure of the Quad model is depicted as a processing tree in Figure 1. In the tree, each path represents a likelihood. Processing parameters with lines leading to them are conditional upon all preceding parameters. For example, according to the model, Overcoming Bias (OB) is conditional upon both activation of associations (AC) and Detection (D). Similarly, Guessing (G) is conditional upon the lack of Activation of associations (1-AC) and the lack of Detection (1-D). The conditional relationships described by the model form a system of equations that predict the number of correct and incorrect responses in different conditions (e.g., compatible and incompatible trials). For example, on an “female/bad” IAT trial, on which a *female name* is presented, the probability of a correct response is [AC × D × OB] + [(1 – AC) × D] + [(1 – AC) × (1 – D) ×(1 – G)] (see Figure 1). This equation sums the three possible paths by which a correct answer will occur. The first part of the equation, AC × D × OB, is the likelihood that the association between *Females* and *Good* is activated and that the correct answer can be detected and that the association is overcome in favor of the detected response. The second part of the equation, (1 – AC) × D, is the likelihood that the association is not activated and that the correct response can be detected. Finally, (1 – AC) × (1 – D) × (1 – G) is the likelihood that the association is not activated and the correct answer cannot be detected and that the participant guesses correctly. The respective equations for each item category (i.e., female names, male names, positive words, and negative words in IAT blocks) are then used to predict the observed proportions of errors in a given data set. The model’s predictions are then compared to the actual data to determine the model’s ability to account for the data. A χ^2^-estimate is computed for the difference between the predicted and observed errors. In order to best approximate the model to the data, the four parameter values are changed through maximum likelihood estimation until they produce a minimum possible value of the χ^2^. The final parameter values that result from this process are interpreted as relative levels of the four processes. The model equations for this study are listed in the **Appendix A**.

Two AC, one OB, one D, and one G parameter were estimated for each group (urban vs. rural) with MultiTree (the error frequencies computed from the raw data for using the Quad model are provided as Supplementary Materials). One AC parameter measured the extent to which associations between “Female” and “Good” were activated in performing the task and the other AC parameter measured the extent to which associations between “Male” and “Bad” were activated in performing the task. The G parameter was coded so that higher scores represent a bias towards guessing with the key for evaluatively positive words. Based on Quad model analyses of other implicit attitudes data with large web-samples, Gonsalkorale et al (2009; 2014) reported error rates of approximately 7%, which is comparable to the error rate reported here (average 8.4%). In such a case, an analysis based on aggregated data is the recommended strategy (Chechile, 2009) because parameter estimates derived from aggregated data are more accurate than parameter estimates derived from each participant separately (e.g., Cohen, Sanborn, & Shiffrin, 2008). Parameter estimates for the two groups are displayed in Table 1. The Quad model fit the data well for the urban group, ⊿χ^2^ (3) = 2.36, *p* = .500. The model also provided a good fit to rural group data, χ^2^ (3) = 4.42, *p* = .220. There were no differences between the urban group and the rural group in estimates of female–good AC, but a marginally significant difference in estimates of male–bad AC, ⊿χ^2^ (1) =3.08, *p* = .079, *w* =.015, indicating that the two groups’ participants had equivalent levels of positive associations with females activated and that urban women had slightly less negative associations with males activated than rural participants. Positive guessing (G) did not differ in groups, ⊿χ^2^ (1) < 2.31. OB was significantly lower for urban women, ⊿χ^2^ (1) = 7.26, *p* = .007, *w* =.023, indicating that urban women less often inhibited their positive associations with females and negative associations with males than were rural women. In addition, there also was a significant difference in detection (D), ⊿χ^2^ (1) = 5.94, *p* = .015, *w* =.021, indicating that urban females were better able to detect correct responses in the IAT task. So, it is not the case that urban women simply have weaker cognitive control across the board; they only seem to have trouble inhibiting attitudinal associations when necessary.

**Table 1:** Parameter Estimates for Gender IAT.

		Estimate [95% Confidence Intervals]	
		
Parameter		Urban women	Rural women

AC	Male/bad	.05 [.03–.07]	.08 [.05–.10]
	Female/good	.06 [.05–.08]	.09 [.06–.11]
OB		.41 [.16–.66]	.81 [.63–.99]
D		.94 [.93–.95]	.92 [.91–.93]
G		.41 [.34–.49]	.51 [.44–.59]

*Note.* AC = Activation of Associations; D = Detection; G = Guessing; OB = Overcoming Bias.

Urban females showed higher pro-female attitudes on IAT performance than did rural females, but they did not possess more pro-female associations. In fact, they even held less negative biased association against men. It seems that the more positive implicit gender attitudes toward women among urban females arise from their diminished inhibition of automatically activated associations.

## Study 2

### Method

**Participants.** Fifty-five female undergraduates at Zhengzhou Normal University participated in the second study. Their mean age was 22.5 years (age range: 17–26 years, 52 right-handed and 3 left-handed). They were recruited via the students’ affairs division and the student union. Following questions relating to age, participants were asked to indicate the location in which they grew up: “In which area did you spend most of your time before entering university?”(c.f., Hinds & Sparks, 2008). Data from 3 participants were discarded due to incomplete responses, leaving 52 participants. Of these, 23 were from urban areas and 29 were from rural areas.

**Materials and Procedure.** The materials and procedure were the same as in the first study.

### Results and Discussion

Replicating the previous study, the overall D effect is –.21, indicating preference for female over male gender. Analysis showed that in-group favoritism was greater for the urban women (*M* = –.39, *SE* = 0.10) than for the rural women (*M* = –.07, *SE* = 0.07), *t* (50) = 2.66, *p* = .011.

The overall error rate for the IAT was 7.34%. Parameter estimates for the two groups are displayed in Table 2 (the error frequency computed from the raw data for using the Quad model are provided as Supplementary Material). The model provided a good fit to the urban group data, ⊿χ^2^ (3) = 1.67, *p* = .639. It also fit the rural group data well, χ^2^ (3) = 2.22, *p* = .532. There were no differences between the urban group and the rural group in estimates of female–good AC, ⊿χ^2^ (1) =.05, *p* = .823 and male–bad AC, ⊿χ^2^ (1) =2.39, *p* = .121. The G parameter and the D parameter did not differ between conditions, all *ps* > .10. However, OB was again significantly lower for urban women, ⊿χ^2^ (1) = 4.36, *p* = .037, *w* =.026.

**Table 2:** Parameter Estimates for Gender IAT in replication study.

		Estimate [Confidence Intervals]	
		
Parameter		Urban women	Rural women

AC	Male/bad	.09 [.05–.12]	.13 [.09–.16]
	Female/good	.08 [.05–.12]	.08 [.04–.11]
OB		.40 [.04–.76]	.81 [.61–1.02]
D		.91 [.89–.93]	.91 [.89–.93]
G		.55 [.44–.65]	.54 [.45–.64]

*Note.* AC = Activation of Associations; D = Detection; G = Guessing; OB = Overcoming Bias.

The results that urban women exhibited more in-group preference than did rural women are replicated in this study. Modeling analyses showed that this effect on IAT performance was associated only with diminished regulation of pro-female and anti-male associations (OB) among the urban women, as indicated by a mini-meta-analysis that combines the data from the 2 studies (Table 3).

**Table 3:** Magnitude of Gender Differences as a Function of measures (K = 2).

Measure	*ES*	95% Conf. Interval	Heterogeneity

IAT effect	0.87**	0.55–1.19	0.28
AC (Female/good)	0.01	0.00–0.02	0.43
AC (Male/bad)	0.02	0.00–0.03	0.10
OB	0.02**	0.01–0.04	0.06
D	0.02	0.00–0.03	1.25
G	0.01	0.00–0.02	0.91

*Note. K* represents the number of effect sizes (*ES*).

** Significance tests of *ES* = 0, *p* < .01.

## General Discussion

Our results revealed that participants have a positive implicit attitude toward women, which replicates the typical effect generally interpreted as a preference for women compared to men (e.g., Nosek & Banaji, 2002). IAT D-scores also suggested that urban women showed more in-group bias than rural women. However, these urban-rural differences in underlying activation towards gender (AC), detection (D), and guessing (G) did not account for reduced bias among rural women. Instead, overcoming bias (OB) differed between rural and urban women in both studies, reflecting lower inhibition of positive associations towards females and negative associations towards males.

This study is the first to independently assess the activation of evaluative associations and self-regulation of gender bias in implicit gender attitudes. When assessed independently, urban and rural women exhibited similar underlying evaluative associations with respect to gender. Compared to rural women, urban women appeared to evaluate their own group more favorably because they were less likely to inhibit their pro-female and anti-male associations. Although women are portrayed more positively in urban China because of changes in culture and urbanization in recent decades, urban women in this study did not exhibit more favorable *in-group associations* based on model analysis, which appears in direct conflict to the IAT-D results. The relatively short history of the feminist movement in urban China may be insufficient to result in more favorable evaluative associations with women than men.

Compared to rural areas, urban areas provide women with egalitarian work opportunities, inclusive education, and pluralistic social roles (Whyte, 2010), which act as cues that may signal favorable responses toward females and result in differences in self-regulatory abilities between urban and rural women. Nevertheless, it is possible that other, unmeasured demographic variables, such as education level may have accounted for the observed pattern of results. Although our study did not provide robust evidence to whether the urban context diminished OB or the rural context enhances it, modeling revealed that differences in behavioral performance on the gender attitude IAT were related to inhibition of in-group biased associations, but not to the strength of associations that were activated. This result goes hand in hand with previous findings based on the Quad model, which indicated that people showed greater implicit bias because they were less likely to regulate the automatic associations they possessed, not because of holding stronger associations in the first place (Gonsalkorale, Sherman & Klauer, 2009). Future research that incorporates detailed demographic variables to validate the current results would be important.

## Competing Interests

The authors declare that they have no competing interests.
